# Supplementary figures and images for: The Effect of Glyceraldehyde-Derived Advanced Glycation End Products on β-Tubulin-Inhibited Neurite Outgrowth in SH-SY5Y Human Neuroblastoma Cells
Source: Nutrients. 2020 Sep 27;12(10):2958. doi: 10.3390/nu12102958 (PMC7601248; doi:10.3390/nu12102958)

A

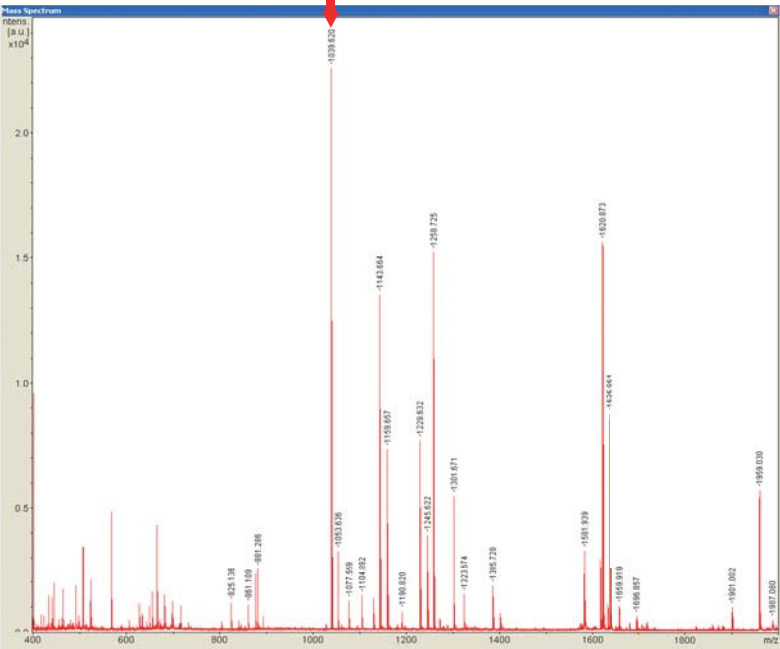

B

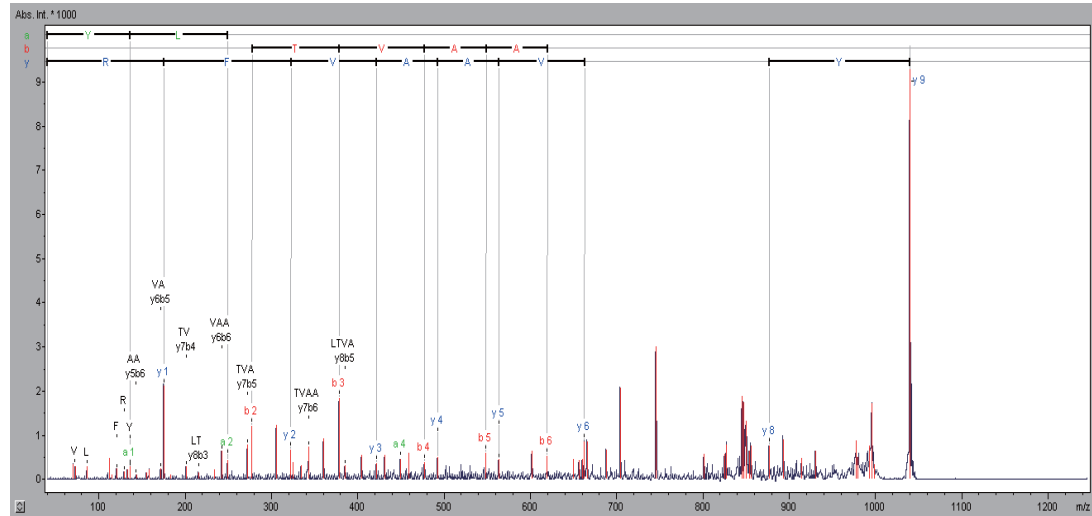

Supplement: Supplementary file 1 [file nutrients-12-02958-s001.pdf]
